# The ionic mechanism of the Purkinje cell dendritic spikes generation and propagation: a model exploration

**DOI:** 10.1186/1471-2202-16-S1-P53

**Published:** 2015-12-18

**Authors:** Yunliang Zang, Erik De Schutter

**Affiliations:** 1 Computational Neuroscience Unit, Okinawa Institute of Science and Technology Graduate University, Onna-son, Okinawa 904-0895, Japan

## 

Due to the lack of sodium channel expression in the Purkinje cell (PC) dendrite, the passively propagated sodium spikes can hardly be detected in the dendrite. However, the substantial expression of T type calcium channels (I_CaT_) and P type calcium channels (I_CaP_) can cause local regenerative spikes to occur in the dendrite after receiving a parallel fiber (PF) stimulus [1].

In this study, with the previously published PC dendrite model [2] from our group as the starting point, we have built a new PC dendrite model incorporating the low threshold A-type K^+ ^current (I_KA_) [3], high threshold activated K^+ ^current (I_KV3_) and hyperpolarization-activated cation current (I_h_). In our model, in agreement with experimental observations [1], the EPSP amplitude increases nonlinearly with the strength of the PF synaptic input until it reaches the spike threshold. During this range, we find that I_CaT _amplifies the EPSP caused by PF synaptic input to boost the EPSP close to the spike threshold. Around the spike threshold there is an obvious spike delay due to the interactive role of I_KA _and I_CaT_. Above the spike threshold, high threshold activated I_CaP _takes over and depolarizes the dendrite rapidly, finally leading to the generation of dendritic spikes. The amplitude of dendritic spikes doesn't increase further with the stimulation intensity and is mainly determined by the struggle among I_CaP_, I_KV3 _and high conductance calcium activated K^+ ^currents (I_BK_). Injecting a negative current can eliminate the PF input evoked dendritic spikes by lowering the membrane potential which implies less activation of the I_CaT _and subsequent less amplification of the EPSP. We also find that the simulated dendritic spikes can't propagate to the whole dendrite due to the regulating role of mainly I_BK _and I_KV3_. Surprisingly by partially blocking I_KV3 _and I_BK _parallel fiber evoked dendritic spikes can propagate to the whole dendrite because of the broadening of its width.

The calcium influx by dendritic spikes is vital to a lot of calcium dependent processes like synaptic plasticity. Understanding the mechanism of generation of the local dendritic spikes evoked by PF stimulus and how their localized nature works will help us better explore how information is processed in the PC dendrite.
